# Comparing Telemedicine and Face-to-Face Consultation Based on the Standard Smoking Cessation Program for Nicotine Dependence: Protocol for a Randomized Controlled Trial

**DOI:** 10.2196/12701

**Published:** 2019-07-09

**Authors:** Tomoyuki Tanigawa, Akihiro Nomura, Maki Kuroda, Tomoyasu Muto, Eisuke Hida, Kohta Satake

**Affiliations:** 1 CureApp Institute Karuizawa Japan; 2 Graduate School of Public Health St Luke's International University Tokyo Japan; 3 Department of Cardiology Kanazawa University Graduate School of Medicine Kanazawa Japan; 4 Innovative Clinical Research Center Kanazawa University Kanazawa Japan; 5 CureApp Inc Tokyo Japan; 6 Department of Biostatistics and Data Science Osaka University Graduate School of Medicine Osaka Japan

**Keywords:** tobacco use disorder, smoking cessation, telemedicine, smartphone, mobile apps, videoconferencing, digital therapeutics, adult, human, randomized controlled trials as topic

## Abstract

**Background:**

Smoking is a major public health concern. In Japan, a 12-week standard smoking cessation support program is available, however, its required face-to-face visits are a key obstacle in completing the program. Telemedicine is a useful way to provide medical treatment at a distance. Although telemedicine for smoking cessation using an internet-based video system has the potential for ensuring better clinical outcomes for patients with nicotine dependence, its efficacy is unclear.

**Objective:**

The aim of this study is to determine the efficacy and feasibility of a smoking cessation support program using an internet-based video system compared with a face-to-face program among patients with nicotine dependence.

**Methods:**

This study will be a randomized, controlled, open-label, multicenter trial. Participants randomized to the intervention arm will undergo an internet-based smoking cessation program, whereas control participants will undergo a standard face-to-face program. We will use the *CureApp Smoking Cessation* (CASC) for both arms, which consists of the CASC smartphone app for patients and a Web-based patient information management system for clinicians with a mobile carbon monoxide checking device. The primary endpoint will be the continuous abstinence rate (CAR) from weeks 9 to 12. Secondary endpoints will be: (1) the smoking cessation success rate at 4, 8, 12, and 24 weeks; (2) CAR from weeks 9 to 24; (3) changes in scores on the mood and physical symptoms scale and 12-Item French Version Of The Tobacco Craving Questionnaire; (4) Kano Test for Social Nicotine Dependence scores at 8, 12, and 24 weeks; (5) time to first lapse after the first visit; (6) nicotine dependence and cognition scale scores at 12 and 24 weeks; (7) usage rate of the CASC; (8) qualitative questionnaire about the usability and acceptability of telemedicine; and (9) presence of product problems or adverse events.

**Results:**

We will recruit 114 participants who are nicotine-dependent but otherwise healthy adults from March to July 2018 and follow up with them until January 2019 (24 weeks). We expect all study results to be available by the end of March 2019.

**Conclusions:**

This will be the first randomized controlled trial to evaluate the efficacy and feasibility of an internet-based (telemedicine) smoking cessation support program relative to a face-to-face program among patients with nicotine dependence. We expect that the efficacy of the telemedicine smoking cessation support program will not be clinically worse than the face-to-face program. If this trial demonstrates that telemedicine does not have clinically worse efficacy and feasibility than a conventional face-to-face program, physicians can begin to offer a more flexible smoking cessation program to patients who may otherwise give up on trying such programs.

**Trial Registration:**

University Hospital Medical Information Network Clinical Trials Registry: UMIN000031620; https://upload.umin.ac.jp/cgi-open-bin/ctr_e/ctr_view.cgi?recptno=R000035975

**International Registered Report Identifier (IRRID):**

DERR1-10.2196/12701

## Introduction

### Background and Research Question

Smoking is a major public health concern responsible for a diverse range of diseases, such as cancers, heart disease, cerebrovascular disease, and chronic obstructive pulmonary disease [1]. In Japan, the estimated number of smokers is more than 20 million, and smoking accounts for about 129,000 deaths per year; the greatest extrinsic cause of death among noninfectious diseases [2]. Furthermore, the excess medical expenses imposed by smoking may be as high as 1.5 trillion yen (approximately US $13 billion) [3]. As such, efforts to reduce the prevalence of smoking would not only cut medical costs, but also help prevent the abovementioned life-threatening diseases [4].

A smoking cessation support program is widely available in Japan for patients with nicotine dependence. This program is agreed to be a standard program by related academic societies and reimbursed by national health insurance [5]. This 12-week program mainly consists of face-to-face counseling with a primary care physician, checking exhaled carbon monoxide (CO) concentration, and prescribing smoking cessation medications such as varenicline or nicotine replacement therapies [6,7]. The national survey report on efficacy of nicotine dependence treatment conducted by the Ministry of Health, Labor, and Welfare of Japan showed that there was a linear relationship between the number of patients’ visits to the institution or clinic and the treatment success rate. Although the primary care physicians made efforts to have patients complete the 12-week program, as few as 29.81% (390/1308) of the participants could do so [8]. However, the preliminary report showed that as many as 75% of 225 participants could complete the modified 8-week-smoking cessation program when conducted as telemedicine, which was a relatively high completion rate compared with a historical control of 50.79% (1763/3471) completion rate at 8 weeks [9]. This result suggested that telemedicine might have the potential to dramatically improve the completion rate of the standard smoking cessation program.

Recently, telemedicine—or the delivery of health care by remote health care providers via communication technologies—has come to be regarded as a useful method of providing medical care to patients. Telemedicine minimizes a patient’s burden of traveling to a health institution and waiting for consultations with their physicians. In addition, it can lead to improved clinical outcomes for patient quality of life [10] and overall quality of care, which include aspects such as efficiency and patient satisfaction [11].

Telemedicine might also be suitable for delivering a smoking cessation support program, as 71.7% of the patients with nicotine dependence in Japan are under 60 years of age and 66.7% of patients are men, both of who are demographics who are unwilling to spend half their day visiting a clinic for treatment [8]. A well-designed randomized trial of 5800 smokers in the United Kingdom demonstrated that a remote smoking cessation program using motivational text messages and customized behavioral change support could double the quit rate compared with the control group who received short text messages unrelated to smoking cessation [12]. Significant improvement of continuous abstinence rate (CAR) at 6 months in the intervention group was reported, with the intervention group improving by 10.7% vs the control group only improving by 4.9%. Moreover, a systematic review showed that interactive and tailored internet-based interventions for smoking cessation might have a moderate effect compared with nonactive controls [13]. Nowadays, with the advent of smartphone technology, apps, and increasing bandwidth, this has allowed health care providers to interact with patients through real-time video-based communication rather than via text- or audio-based communication [14]. Video-based telemedicine systems can be cost-saving, promote more active self-care, and result in better clinical outcomes in the management of various diseases [15]. For example, researchers of home health care in the United States reported, based on a randomized control trial, that video communication technology could improve the quality indicators of the practice and showed cost effectiveness when it could substitute in-person clinical visits [16]. Another randomized clinical trial suggested that video-based telemedicine specialist consultation was well accepted and cost-effective among nonacute headache patients [17]. Other, previous research showed that video consultation, added to standard care, could improve the glycemic control of diabetes patients who did not respond to standard care [18].

The *CureApp Smoking Cessation* (CASC) is a novel smartphone app paired with a mobile CO checking device that was developed by CureApp Inc, Tokyo, Japan, to improve treatment success of the smoking cessation program for patients with nicotine dependence [19,20]. CASC can provide patients with accurate knowledge of nicotine dependency and tips for changing their behavior, as well as help them monitor their own exhaled CO levels with a personal mobile CO checking device at home. They can then share these data with their primary care physicians remotely. A prospective, single-arm, pilot study demonstrated that a smoking cessation program with a CASC group showed a higher CAR from weeks 9 to 24 than those without a CASC group [21].

Therefore, telemedicine might have the potential to enhance the smoking cessation success rate and other clinical outcomes among patients with nicotine dependence by helping them achieve easier access to smoking cessation programs. However, it remains unclear whether the smoking cessation program using an internet-based video system is effective and safe relative to a conventional face-to-face program.

### Objectives

This is a proof-of-concept study aiming to determine the efficacy and feasibility of a smoking cessation support program using an internet-based video system compared with a face-to-face program, both of which use the CASC, among patients with nicotine dependence.

## Methods

### Study Design

**Figure 1:**
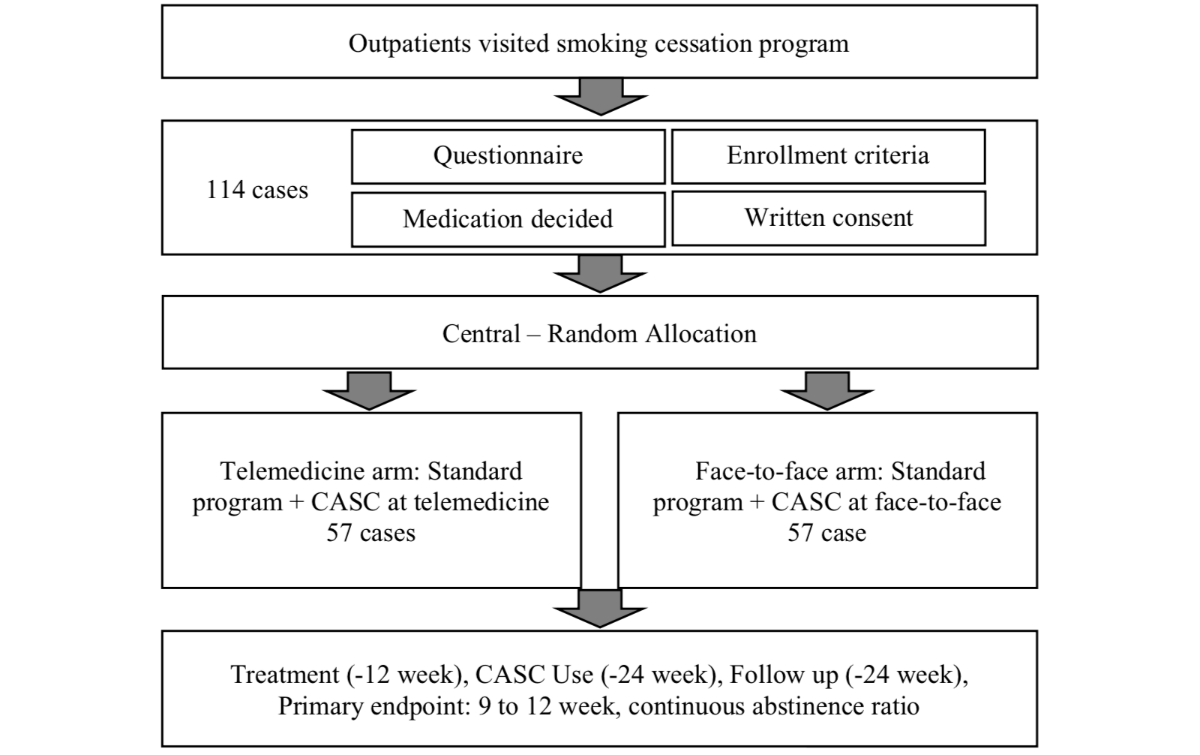
Scheme of this trial protocol. CASC: CureApp Smoking Cessation.

This study will be a randomized, controlled, open-label, multicenter trial. The study will be conducted in 4 community clinics located in Tokyo, Japan: Tokyo-Eki Center-building Clinic, Shinjuku Research Park Clinic, Fukuwa Clinic and Miyazaki RC Clinic. Figure 1 provides an outline of the trial.

Participants in both arms will undergo the smoking cessation support program used in Japan. For the telemedicine arm, the entire program will be conducted remotely via an internet-based video system except for the first registration visit [22], whereas participants in the control arm will follow the standard program in the face-to-face manner. Participants in both arms will be asked to download the CASC smartphone app as well as be given a mobile CO checker. These will be used for 24 weeks. The follow-up schedule is shown in Table 1.

### Participants

We will recruit nicotine-dependent but otherwise healthy adults from March to July 2018. We plan to follow them up until January 2019 (ie, for 24 weeks). We will include the participants who are diagnosed as nicotine dependent, have smoking history of Brinkman index (BI) >200, and have a determination to quit smoking immediately. Then, we will exclude those participants with severe mental illness and those with any smoking cessation aid, besides prescribed medication at clinics before or after enrollment, based on the subjective questionnaire on the enrollment visit (detail of the inclusion criteria is shown in Multimedia Appendix 1, and the exclusion criteria is shown in the Multimedia Appendix 2). Not-Burning-Tobacco product users can be included if the participant meets the inclusion criteria of the BI, which was defined as the number of combustible traditional tobacco packs per day multiplied by the number of smoking years. Primary doctors at each study site will obtain written informed consent from all study participants, and the consent forms that will be used have been approved by our institutional review board. We will inform all participants that their medical care will not be affected if they refuse to be enrolled in the trial. In addition, participants will be able to drop out of the study at any time. Clinics participating in this study can provide the smoking cessation support program and have the necessary equipment to conduct telemedicine (eg, Wi-Fi access in the facility).

**Table 1:** Assessment and evaluation schedule of the study.

Variables	Registration	Observation period					
Assessments	Day 1	Day 15 (2 weeks)	Day 29 (4 weeks)	Day 57 (8 weeks)	Day 85 (12 weeks)	Day 168 (24 weeks)	At withdrawal
Patients’ profile	✓^a^	—^b^	—	—	—	—	—
Tobacco Dependence Screening Test	✓	—	—	—	—	—	—
Brinkman Index	✓	—	—	—	—	—	—
Fagerström test for nicotine dependence	✓	—	—	—	—	—	—
12-item French version of the Tobacco Craving Questionnaire	✓	✓	✓	✓	✓	✓	—
Kano Test for Social Nicotine Dependence	✓	—	—	✓	✓	✓	—
Mood and physical symptoms scale	✓	✓	✓	✓	✓	✓	—
Nicotine dependence and cognition scale	✓	—	—	—	✓	✓	—
Exhaled carbon oxide concentration	✓	✓	✓	✓	✓	✓	✓
Smoking status	✓	✓	✓	✓	✓	✓	✓
Device use status	—	✓	✓	✓	✓	✓	✓
Adverse events	—	—	—	—	—	—	—

^a^Information collected.

^b^Not applicable.

### Sample Size

As shown in the findings of a previous pilot study of the CASC, the difference in CAR from weeks 9 to 12 between using a CASC smartphone app group (78%) and historical control groups (not using the application; 54%) is 24% [21,23]. Accordingly, we assumed that the treatment effect of both arms using the CASC in the current study will be 80% and the threshold amount should be 15%, which is smaller than the difference between the CASC group and historical control group in the previous study. The sample size was calculated as 114 (57 per arm) based on the precision of the estimate that the lower limit of the 95% CI of the difference between treatment effects exceeded the threshold amount of 15%. Therefore, we aim to recruit at least 114 participants to allow for this sample size.

### Randomization

For randomization, we will use the block randomization method (with 4 blocks) with a 1:1 allocation ratio. Participants will be allocated in this way to either the intervention (telemedicine) or control (face-to-face) arm. The randomization will be performed at the time of participants’ registration by a staff member using a computer-generated random sequence for each participating clinic.

### Interventions and Control

Participants randomized to the intervention (ie, telemedicine) arm will receive an internet-based support program for smoking cessation using the CASC. In contrast, participants randomized to the control (face-to-face) arm will receive the conventional face-to-face standard smoking cessation support program, also using the CASC.

The standard smoking cessation support program in Japan consists of 5 clinic visits over 12 weeks, including doctor consultation and exhaled CO check at a registered institution or clinic [5]. All study participants will meet their primary care physicians face to face during the first visit to ensure that they fully understand the study protocol. On this first visit, the physicians will decide on the appropriate treatment drug, provide guidance in accordance with the standard program procedure, and provide participants with the CASC. Following the first visit, control patients are supposed to visit the clinic in weeks 2, 4, 8, and 12, and at each visit, their primary doctors will administer the treatment drug.

Patients in the telemedicine arm will undergo the standard smoking cessation support program with the CASC. However, instead of visiting the clinics, they will meet their physicians to receive counseling via a video-conference system and a standardized telemedicine platform app, in accordance with the guideline for telemedicine in Japan [22]. As in the standard procedure, they will meet with their physicians via internet at each of the planned visits (2, 4, 8, and 12 weeks).

The CASC consists of the following: (1) a CASC smartphone app for patients with nicotine dependence, (2) a mobile CO checker for patients, and (3) a Web-based patient management software for health care providers [20]. Participants are asked to watch a 5- to 10-min lecture on smoking cessation every day (see Multimedia Appendix 3 for the specifics). They are also asked to write a short comment as a diary and to check their exhaled CO concentration with the device. Primary care physicians can monitor their diaries, physical conditions, lecture progress, and exhaled CO concentration data.

The CASC will be delivered by the study sponsor, CureApp Inc, Tokyo, Japan, and will be carefully managed to avoid being used outside the current study at each institution or clinic. A prescription code specific to each practicing institution or clinic is required to activate the entire system issued by the sponsor, and the sponsor regularly (at least once a year) performs an inventory and confirms the devices are not used outside the research.

### Endpoints

The primary endpoint of this study will be the CAR from weeks 9 to 12. We hypothesize that the telemedicine group will not show clinically worse CAR from weeks 9 to 12 than the control group (threshold of 15%). The CAR is defined as the percentage of individuals continuously not smoking during the specified period. We will also evaluate the following secondary endpoints: (1) smoking cessation success rate at the points of 4, 8, 12, and 24 weeks; (2) CAR from weeks 9 to 24; (3) changes in the scores on the mood and physical symptoms scale (MPSS) [24] and 12-item French version of the Tobacco Craving Questionnaire (FTCQ-12) [25]; (4) Kano Test for Social Nicotine Dependence (KTSND) score [26] at 8, 12, and 24 weeks; (5) time to first lapse after the first visit; (6) nicotine dependence and cognition scale (NDCS) score at 12 and 24 weeks; (7) usage rate of the CASC; (8) qualitative questionnaire about the usability and acceptability of telemedicine; and (9) the presence of product problems or adverse events.

The NDCS is a scale for measuring nicotine dependence and cognitive impairment among smokers. It consists of 8 items, each rated on a 4-point scale (ranging from 0 to 3). A higher total score (minimum=0, maximum=24) implies more severe dependency or cognitive impairment.

### Follow-Up Schedule and Data Collection

The follow-up schedule is shown in Table 1. Follow-up visits will be conducted in each clinic. At the registration visit, we will record patients’ baseline profile, Tobacco Dependence Screening Test [27], BI, Fagerström test for nicotine dependence [28], FTCQ-12, KTSND, MPSS, NDCS, exhaled CO concentration, and smoking cessation status. The baseline profile will consist of age, gender, body weight, years of smoking, number of cigarettes per day, and past medical history. We will check their FTCQ-12, KTSND (after 8 weeks), MPSS, NDCS (after 12 weeks), exhaled CO concentration, smoking status, the frequency of application usage and presence of product problems, or adverse events at 2 weeks (±1 week), 4 weeks (±1 week), 8 weeks (±2 weeks), 12 weeks (±2 weeks), and 24 weeks (±4 weeks). The qualitative questionnaire about the usability and acceptability of telemedicine will be asked only for the participants in the telemedicine arm at 24 weeks. Each doctor at the study site will be responsible for the case report form, and the monitoring staff, who are employees of the sponsor, will visit each study site and check the data quality during the study period.

### Statistical Methods

We will compare all endpoints between the telemedicine and the face-to-face arms. Baseline characteristics will be described by means and standard deviations, or medians and interquartile ranges (for continuous variables), or proportions (for categorical variables). We will analyze the primary outcome using the full analysis set (excluding participants who violate the abovementioned inclusion or exclusion criteria). For all outcomes, summary statistics and group difference measures (eg, odds ratios and mean differences) will be presented with 95% CIs and *P* values from 2-sided tests (eg, logistic regression and analysis of covariance).

### Patient and Public Involvement

Participants in this study were not involved in the design, recruitment, conduct, or assessment of the study.

### Ethics and Dissemination

We will conduct this study in compliance with the Declaration of Helsinki, Medical Device Good Clinical Practice guidelines, and all other applicable laws and guidelines in Japan. This protocol and related documents of all the participating clinics were approved by the Tokyo-Eki Center-building clinic institutional review board. We will always use the latest version of the approved documents. We used the SPIRIT reporting guidelines for submitting this protocol to the journal [29]. We will disseminate the results at national or international conferences and in a peer-reviewed journal.

## Results

As of November 2018, 115 participants with nicotine-dependency were already recruited and will be followed up until January 2019 (24 weeks). We expect all study results to be available by the end of March 2019.

## Discussion

### Rationale

This will be the first randomized control trial to evaluate the efficacy and feasibility of an internet-based video-assisted smoking cessation support program compared with a face-to-face program in patients with nicotine dependence. We expect that the efficacy of the telemedicine smoking cessation support program (in terms of CAR from weeks 9 to 12) will not be clinically worse than the face-to-face program.

Recent advances in mobile technologies have dramatically changed nearly every aspect of our daily lives. Internet services and smartphone apps play a particularly key role because they help us directly communicate with each other even when apart. Nowadays, smartphone and internet-based video communication have been considered useful tools for improving accessibility to smoking cessation programs, thereby improving the outcomes of patients with nicotine dependence. Scott-Sheldon et al demonstrated in their meta-analysis that a mobile phone short messaging service intervention led to substantial benefits for smoking cessation [30]. Moreover, Carlson et al reported that a group smoking cessation program delivered via video-conference technology in a rural area of Canada was as effective as an in-person group program conducted in an urban area [31]. Thus, we expect that our internet-based smoking cessation support program will have the potential to be comparable to the conventional face-to-face program because it can empower the patients to more conveniently access the relevant medical information and health care providers.

### Study Design

Considering that the aim of this study is to compare different ways of delivering a standard 12-week smoking cessation program, a CAR for the last 4 weeks of the treatment period would be the ideal endpoint. It would also be appropriate to compare those 2 groups for CAR from weeks 9 to 12 because a previous phase III clinical trial on smoking cessation aid medications, such as varenicline, evaluated CAR for the same period to assess the efficacy [32].

In this study, we will use the CASC for both arms as it serves to complement the standard smoking cessation program. A previous single arm, prospective pilot study showed that the CASC, in addition to the standard program, led to significantly higher CAR from weeks 9 to 24 when compared with a historical control group [21]. Currently, there is an ongoing Phase III randomized controlled clinical trial to evaluate the efficacy of the CASC [20]. The standard smoking cessation program in Japan requires that patients’ exhaled CO concentration be measured at each clinic visit [5], and the information on their CO level is helpful for the primary doctor to give treatment advice on each visit. The CO checker in the CASC enables all patients—even those in the telemedicine group—to monitor their own exhaled CO concentration level at home in the validated way. Therefore, we believe that the CASC must be used in both arms to fairly evaluate the difference between the telemedicine and face-to-face treatment programs.

### Limitations

This study has several limitations. First, this study will be conducted in multiple centers, but participants will be limited to residents in an urban area in Tokyo, Japan, which may limit the generalizability of the study findings. We might need to conduct further investigations to apply the study findings to other cohorts. Second, participants in both groups are going to use the CASC, but this system has not yet been cleared by the Pharmaceuticals and Medical Devices Agency. Therefore, the outcomes for each group must be carefully interpreted because they might be the result of mixed effects of the study intervention and the CASC on the standard smoking cessation program. Nevertheless, since the program in Japan requires evaluation of exhaled CO concentration at each visit, supplying participants with the CASC is the only realistic and validated solution to conduct this study at present. Third, the primary physicians can prescribe treatment drugs, either varenicline or nicotine patch, to support the patients in quitting smoking according to a physician’s discretion. Although the institution is an allocation factor for equal randomization, the baseline characteristics of the drug assignment might not be matched between the 2 arms, which could cause the biased results.

### Conclusions

In conclusion, we will test whether an internet-based smoking cessation support program does not have clinically worse efficacy and feasibility than a face-to-face smoking cessation program among patients with nicotine dependence. If we obtain the expected findings, physicians can offer a more flexible standard smoking cessation program to patients who might otherwise give up on the idea of trying such programs. In addition, this study could be a milestone to expand the scope of the effective smoking cessation support program, which might contribute to an improvement of public health and reduction of medical expenses.

## Appendix

Multimedia Appendix 1 Inclusion criteria.

Multimedia Appendix 2 Exclusion criteria.

Multimedia Appendix 3 List of smoking cessation treatment lectures provided by the application.

## Abbreviations

BI: Brinkman index
CAR: Continuous abstinence rate
CASC: CureApp Smoking Cessation
CO: carbon monoxide
FTCQ-12: 12-item French version of the Tobacco Craving Questionnaire
KTSND: Kano Test for Social Nicotine Dependence
MPSS: mood and physical symptoms scale
NDCS: nicotine dependence and cognition scale

## References

[1] Ikeda N, Inoue M, Iso H, Ikeda S, Satoh T, Noda M, Mizoue T, Imano H, Saito E, Katanoda K, Sobue T, Tsugane S, Naghavi M, Ezzati M, Shibuya K (2012). Adult mortality attributable to preventable risk factors for non-communicable diseases and injuries in Japan: a comparative risk assessment. PLoS Med.

[2] Ikeda N, Saito E, Kondo N, Inoue M, Ikeda S, Satoh T, Wada K, Stickley A, Katanoda K, Mizoue T, Noda M, Iso H, Fujino Y, Sobue T, Tsugane S, Naghavi M, Ezzati M, Shibuya K (2011). What has made the population of Japan healthy?. Lancet.

[3] Nakamura M (2017). Report of the Health Labour Sciences Research Grant (09004A).

[4] Igarashi A, Goto R, Suwa K, Yoshikawa R, Ward AJ, Moller J (2016). Cost-effectiveness analysis of smoking cessation interventions in Japan using a discrete-event simulation. Appl Health Econ Health Policy.

[5] (2014). The Japanese Circulation Society.

[6] Gonzales D, Rennard SI, Nides M, Oncken C, Azoulay S, Billing CB, Watsky EJ, Gong J, Williams KE, Reeves KR, Varenicline Phase 3 Study Group (2006). Varenicline, an alpha4beta2 nicotinic acetylcholine receptor partial agonist, vs sustained-release bupropion and placebo for smoking cessation: a randomized controlled trial. J Am Med Assoc.

[7] Hartmann-Boyce J, Chepkin SC, Ye W, Bullen C, Lancaster T (2018). Nicotine replacement therapy versus control for smoking cessation. Cochrane Database Syst Rev.

[8] (2017). Ministry of Health, Labour and Welfare, Japan.

[9] Kitada M (2017). Evaluation on the Impact of Telemedicine for Clinic Visits and Treatment Success of Nicotine Dependence.

[10] Flodgren G, Rachas A, Farmer AJ, Inzitari M, Shepperd S (2015). Interactive telemedicine: effects on professional practice and health care outcomes. Cochrane Database Syst Rev.

[11] Schwamm LH, Chumbler N, Brown E, Fonarow GC, Berube D, Nystrom K, Suter R, Zavala M, Polsky D, Radhakrishnan K, Lacktman N, Horton K, Malcarney M, Halamka J, Tiner AC, American Heart Association Advocacy Coordinating Committee (2017). Recommendations for the implementation of telehealth in cardiovascular and stroke care: a policy statement from the American heart association. Circulation.

[12] Free C, Knight R, Robertson S, Whittaker R, Edwards P, Zhou W, Rodgers A, Cairns J, Kenward MG, Roberts I (2011). Smoking cessation support delivered via mobile phone text messaging (txt2stop): a single-blind, randomised trial. Lancet.

[13] Taylor GM, Dalili MN, Semwal M, Civljak M, Sheikh A, Car J (2017). Internet-based interventions for smoking cessation. Cochrane Database Syst Rev.

[14] Clay-Williams R, Baysari M, Taylor N, Zalitis D, Georgiou A, Robinson M, Braithwaite J, Westbrook J (2017). Service provider perceptions of transitioning from audio to video capability in a telehealth system: a qualitative evaluation. BMC Health Serv Res.

[15] Wade VA, Karnon J, Elshaug AG, Hiller JE (2010). A systematic review of economic analyses of telehealth services using real time video communication. BMC Health Serv Res.

[16] Johnston B, Wheeler L, Deuser J, Sousa KH (2000). Outcomes of the Kaiser permanente tele-home health research project. Arch Fam Med.

[17] Müller KI, Alstadhaug KB, Bekkelund SI (2016). Acceptability, feasibility, and cost of telemedicine for nonacute headaches: a randomized study comparing video and traditional consultations. J Med Internet Res.

[18] Hansen CR, Perrild H, Koefoed BG, Zander M (2017). Video consultations as add-on to standard care among patients with type 2 diabetes not responding to standard regimens: a randomized controlled trial. Eur J Endocrinol.

[19] Masaki K, Tateno H, Kameyama N, Satake K, Suzuki S, Muto T (2017). Preliminary Report of a Smartphone Application? CureApp for Smoking Cessation.

[20] Nomura A, Tateno H, Masaki K, Muto T, Suzuki S, Satake K, Hida E, Fukunaga K (2019). A novel smoking cessation smartphone app integrated with a mobile carbon monoxide checker for smoking cessation treatment: protocol for a randomized controlled trial. JMIR Res Protoc.

[21] Masaki K, Tateno H, Kameyama N, Morino E, Watanabe R, Sekine K, Ono T, Satake K, Suzuki S, Nomura A, Betsuyaku T, Fukunaga K (2019). Impact of a novel smartphone app (CureApp Smoking Cessation) on nicotine dependence: prospective single-arm interventional pilot study. JMIR Mhealth Uhealth.

[22] (2018). Ministry of Health, Labour and Welfare.

[23] (2008). Ministry of Health, Labour and Welfare.

[24] West R, Hajek P (2004). Evaluation of the mood and physical symptoms scale (MPSS) to assess cigarette withdrawal. Psychopharmacology (Berl).

[25] Berlin I, Singleton EG, Heishman SJ (2010). Validity of the 12-item French version of the Tobacco Craving Questionnaire in treatment-seeking smokers. Nicotine Tob Res.

[26] Otani T, Yoshii C, Kano M, Kitada M, Inagaki K, Kurioka N, Isomura T, Hara M, Okubo Y, Koyama H (2009). Validity and reliability of Kano Test for Social Nicotine Dependence. Ann Epidemiol.

[27] Kawakami N, Takatsuka N, Inaba S, Shimizu H (1999). Development of a screening questionnaire for tobacco/nicotine dependence according to ICD-10, DSM-III-R, and DSM-IV. Addict Behav.

[28] Heatherton TF, Kozlowski LT, Frecker RC, Fagerström KO (1991). The Fagerström Test for Nicotine Dependence: a revision of the Fagerström Tolerance Questionnaire. Br J Addict.

[29] Chan A, Tetzlaff JM, Altman DG, Laupacis A, Gøtzsche PC, Krleža-Jerić K, Hróbjartsson A, Mann H, Dickersin K, Berlin JA, Doré CJ, Parulekar WR, Summerskill WSM, Groves T, Schulz KF, Sox HC, Rockhold FW, Rennie D, Moher D (2013). SPIRIT 2013 statement: defining standard protocol items for clinical trials. Ann Intern Med.

[30] Scott-Sheldon LA, Lantini R, Jennings EG, Thind H, Rosen RK, Salmoirago-Blotcher E, Bock BC (2016). Text messaging-based interventions for smoking cessation: a systematic review and meta-analysis. JMIR Mhealth Uhealth.

[31] Carlson LE, Lounsberry JJ, Maciejewski O, Wright K, Collacutt V, Taenzer P (2012). Telehealth-delivered group smoking cessation for rural and urban participants: feasibility and cessation rates. Addict Behav.

[32] Nakamura M, Oshima A, Fujimoto Y, Maruyama N, Ishibashi T, Reeves KR (2007). Efficacy and tolerability of varenicline, an alpha4beta2 nicotinic acetylcholine receptor partial agonist, in a 12-week, randomized, placebo-controlled, dose-response study with 40-week follow-up for smoking cessation in Japanese smokers. Clin Ther.

