# Supplementary material for: Comparing Telemedicine and Face-to-Face Consultation Based on the Standard Smoking Cessation Program for Nicotine Dependence: Protocol for a Randomized Controlled Trial
Source: JMIR Res Protoc. 2019 Jul 9;8(7):e12701. doi: 10.2196/12701 (PMC6647761; doi:10.2196/12701)
Supplement: Multimedia Appendix 1 [file resprot_v8i7e12701_app1.pdf]

Supplemental Table 1. Inclusion criteria

We included participants who met all the following criteria:
1) Diagnosed with nicotine dependent (TDS ≥5 points).
2) Had a Brinkman index ≥200.
3) Had will to quit smoking immediately.
4) Agreed to undergo the smoking cessation treatment program.
5) Could use a smartphone (Operating System (OS): Android® 6.0 and above, or iOS® 10.0 and above).

Abbreviations: TDS, Tobacco Dependence Screening test.
Brinkman index = number of tobacco pack per day multiplied by number of smoking years
