# Supplementary material for: Comparing Telemedicine and Face-to-Face Consultation Based on the Standard Smoking Cessation Program for Nicotine Dependence: Protocol for a Randomized Controlled Trial
Source: JMIR Res Protoc. 2019 Jul 9;8(7):e12701. doi: 10.2196/12701 (PMC6647761; doi:10.2196/12701)
Supplement: Multimedia Appendix 2 [file resprot_v8i7e12701_app2.pdf]

Supplemental Table 2. Exclusion criteria

We excluded participants with any of the following criteria:

1) Had severe mental illness.
2) Could not visit the follow-up clinics for six months
3) Had used smoking cessation supplements or medication before the registration.
4) Planned to use any smoking cessation aids or to participate in any kind of smoking cessation activities (not limited to smoking cessation therapy) during the study.
5) Visited clinics regularly for diseases other than nicotine dependence within 12 weeks after registration.
