# Supplementary material for: Comparing Telemedicine and Face-to-Face Consultation Based on the Standard Smoking Cessation Program for Nicotine Dependence: Protocol for a Randomized Controlled Trial
Source: JMIR Res Protoc. 2019 Jul 9;8(7):e12701. doi: 10.2196/12701 (PMC6647761; doi:10.2196/12701)
Supplement: Multimedia Appendix 3 [file resprot_v8i7e12701_app3.pdf]

Supplemental Table 3. List of smoking cessation treatment lectures provided by the application

No.	Lecture title
1	Behavioral contract
2	Nicotine dependence is a disease
3	Nicotine dependence has two components<br>One: physical dependence
4	Nicotine dependence has two components<br>Two: psychological dependence
5	Build a smoke-free environment
6	Change your behavior patterns
7	Find something else rather than smoking
8	Declaration of smoking cessation
9	Withdrawal symptoms
10	Smoking cessation techniques: check-ups
11	Keep making an effort for smoking cessation
12	Self-assertiveness workshop
13	Praise yourself when you succeed
14	Visit the clinic more often, and your will gain higher success
15	Body weight may increase temporally, while smoking cessation is much better for your health
16	Positive impact of smoking cessation on beauty
17	Build a perception that you may fail to stop smoking
18	Decline the call for smoking together by friends
19	It's not because you are weak-willed that you cannot quit smoking
20	Smoking even one piece of cigarette resets all your efforts for smoking cessation
21	You cannot relieve your stress through smoking
22	Relapse prevention training
23	Control your smoking mind
24	You reach the middle of the program
25	Graduation (deployed at the completion of the 5th outpatient-clinic visit)
26–29	Prevention program for relapse of smoking
30	Final chapter
